# Relationship Between Short-chain Fatty Acids and Parkinson’s Disease: A Review from Pathology to Clinic

**DOI:** 10.1007/s12264-023-01123-9

**Published:** 2023-09-27

**Authors:** Wen-Xiang Duan, Fen Wang, Jun-Yi Liu, Chun-Feng Liu

**Affiliations:** 1 Department of Neurology and Clinical Research Center of Neurological Disease, The Second Affiliated Hospital of Soochow University, Suzhou, 215004 China; 2 Department of Neurology, Dushu Lake Hospital affiliated to Soochow University, Suzhou, 215125 China; 3 Jiangsu Key Laboratory of Neuropsychiatric Diseases and Institute of Neuroscience, Soochow University, Suzhou, 215123 China

**Keywords:** Parkinson’s disease, Short-chain fatty acids, Microbial metabolites, Brain-gut-microbiota axis

## Abstract

Parkinson’s disease (PD) is a complicated neurodegenerative disease, characterized by the accumulation of α-synuclein (α-syn) in Lewy bodies and neurites, and massive loss of midbrain dopamine neurons. Increasing evidence suggests that gut microbiota and microbial metabolites are involved in the development of PD. Among these, short-chain fatty acids (SCFAs), the most abundant microbial metabolites, have been proven to play a key role in brain-gut communication. In this review, we analyze the role of SCFAs in the pathology of PD from multiple dimensions and summarize the alterations of SCFAs in PD patients as well as their correlation with motor and non-motor symptoms. Future research should focus on further elucidating the role of SCFAs in neuroinflammation, as well as developing novel strategies employing SCFAs and their derivatives to treat PD.

## Introduction

Parkinson’s disease (PD) is the second most common neurodegenerative disease in the world, causing huge health and economic burdens on society. According to estimates, the frequency of PD will increase as the population ages and the mortality risk for PD patients has risen by 1.5–2.2 times [1, 2]. PD is characterized by classical motor symptoms, including bradykinesia, resting tremor, rigidity, and postural instability. In addition, PD patients are often complicated with many non-motor symptoms, such as constipation, anosmia, cognitive impairment, and depression [3]. Since the first direct demonstration of the close relationship between PD and the gut microbiota using fecal transplantation, the role of intestinal microorganisms and metabolites in the development of PD has been gradually emphasized [4]. Short-chain fatty acids (SCFAs), produced in the colon by the fermentation of dietary fiber, not only play an essential role in maintaining intestinal homeostasis but also affect the function of the central nervous system (CNS) through the peripheral circulation and vagus nerve. A significant reduction in the synthesis of SCFAs has been found in the feces of PD patients, and many animal studies have demonstrated the protective effect of SCFAs against PD. Despite the rapid progress in recent years, there are still no reviews specifically summarizing and discussing these aspects. This review aims to explain the relationship between SCFAs and PD from the perspective of pathology and the clinic based on the existing evidence and put forward suggestions for future research directions.

## Metabolism of SCFAs

SCFAs are organic fatty acids with fewer than six carbon atoms, and mainly include acetic acid (AA), propionic acid (PA), and butyric acid (BA), with a proportion of approximately 60:20:20. The primary source of SCFAs is microbial fermentation of dietary fiber. Microorganisms can also obtain carbohydrates from the colonic mucus layer [5]. After production, SCFAs are quickly absorbed by colon epithelial cells through the monocarboxylate transporter (MCT) and sodium-coupled monocarboxylate transporter (SMCT) [6]. In the colon epithelium, SCFAs can be used in mitochondria β-oxidation and the citric acid cycle to provide energy for epithelial cells [7]. Only 5–10% of SCFAs are excreted through feces, while the unmetabolized SCFAs are delivered to the portal vein circulation. To prevent high SCFAs concentrations in the blood, the liver metabolizes most of the propionate and butyrate in the portal circulation [8]. Finally, only a small amount of acetate, propionate, and butyrate from the colon reaches the systemic circulation and peripheral tissues. Due to the high-fat solubility of SCFAs and the high expression of transporters on endothelial cells, SCFAs entering the circulation can cross the blood-brain barrier (BBB) and reach the brain [9]. For the first time, 14 C-SCFA uptake in the brain was discovered after injection into the carotid artery of rats [10, 11]. A study also reported that the tissue concentration of butyrate in the human brain was 17.0 pmol/mg, and propionate was 18.8 pmol/g [12]. These results suggest a potential role of SCFAs in the brain.

## Mechanism of SCFAs

### Inhibition of Histone Deacetylase

In the nucleus, histone acetylation and histone deacetylation are in dynamic balance and are jointly regulated by histone acetyltransferase and histone deacetylase (HDAC). In neurodegenerative diseases, acetylation homeostasis is significantly disrupted, leading to a decrease in the histone acetylation level. HDACs inhibitors can increase histone acetylation and promote the expression of genes involved in cell survival and neuroprotection [13]. SCFAs can enter cells via transporters and inhibit HDACs activity, among which BA is one of the most effective inhibitors of Class I and Class IIa HDACs. According to the experimental results in various models, HDACs inhibitors have been proven to have neuroprotective effects on PD. In 1-Methyl-4-phenyl-1,2,3,6-tetrahydropyridine (MPTP) induced PD mice, HDACs inhibitors, such as sodium valproate and sodium butyrate, increased the expression of glial cell-derived neurotrophic factor (GDNF) and brain-derived neurotrophic factor (BDNF) in astrocytes, while histone H3 acetylation was significantly increased [14–16]. In rotenone-induced PD drosophila, sodium butyrate effectively reduced dopaminergic neurodegeneration and increased dopamine levels in the brain. In addition, drosophila with HDAC gene knockout can resist rotenone-induced movement disorders and early death [17]. In lipopolysaccharide (LPS)-treated glial cells, HDACs inhibitors can provide neuroprotection by inhibiting the release of proinflammatory cytokines and chemokines from microglia [18, 19]. Therefore, SCFAs can increase the synthesis of neurotrophic factors and suppress the expression of inflammatory factors to inhibit neurodegeneration by inhibiting HDACs.

### Combination with GPCR

**Fig. 1:**
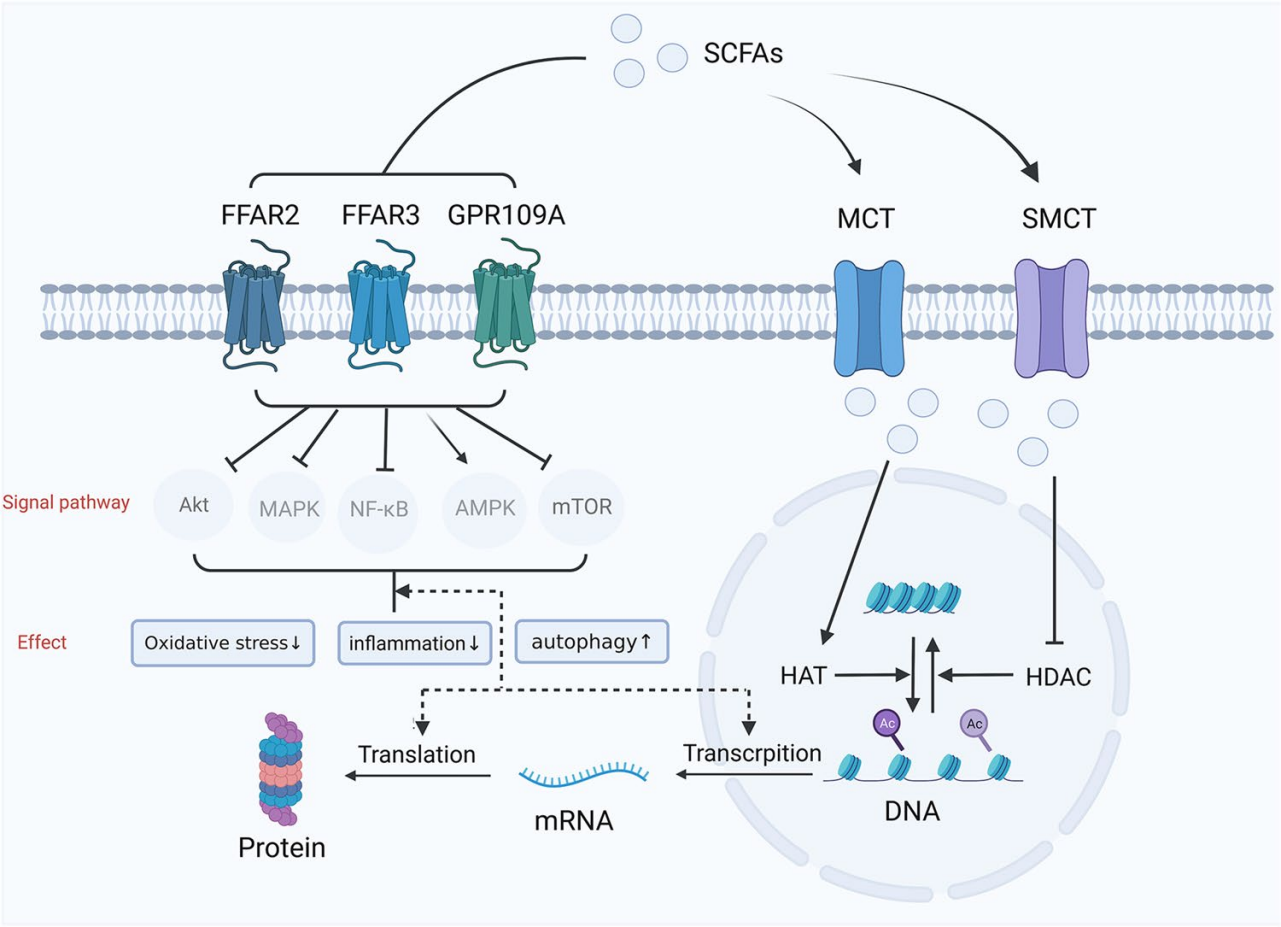
Mechanism of short-chain fatty acids. SCFAs primarily act on target cells through two mechanisms. First, SCFAs can enter the cell through MCT and SMCT transporters on the cell membrane, and then enter the cell nucleus to inhibit HDAC and to activate HAT, resulting in increased histone acetylation, the gradual loosening of dense chromosomes, and finally increased gene expression. Another mode of action is to combine GPCR on the cell membrane, such as FFAR2, FFAR3, or GPR109A, which can inhibit downstream NF-κB, Akt, MAPK, mTOR, and activate downstream AMPK signal pathways, thus regulating gene transcription and translation to alleviate inflammation, reduce oxidative stress and enhance the role of autophagy. NF-κB, nuclear factor-κB; AMPK, adenosine 5’-monophosphate (AMP)-activated protein kinase; MAPK, mitogen-activated protein kinase; mTOR, mammalian target of rapamycin. Created with BioRender.com.

SCFAs can bind to G protein-coupled receptors (GPCRs) on the cell membrane, including GPR41/free fatty acid receptor 3 (FFAR3), GPR43/FFAR2, GPR42, and GPR109a [20]. After binding to these membrane receptors, SCFAs can inhibit downstream NF-κB and MAPK signaling to suppress inflammation, and enhance AMPK signaling while inhibiting mTOR signaling to enhance autophagy and enhance Nfr2 signaling to reduce oxidative stress [21–23]. These complicated signaling pathways regulate cellular immunity, metabolism, and other processes (Fig. 1). FFAR2 has a higher affinity for SCFAs with shorter chains, but FFAR3 has a higher affinity for SCFAs with longer chains, like butyrate. FFAR2 and FFAR3 are abundant in immune cells, adipose tissue, intestine, and bone marrow [24–26]. The expression of FFAR3 in sympathetic ganglia appears essential for controlling sympathetic nerve activity, as demonstrated by reduced activity in FFAR3 knockout mice [27]. Furthermore, the expression of FFAR3 and transporters of SCFAs have been confirmed in brain endothelial cells, indicating that SCFAs may affect the function of the BBB [28].

Although most research on FFAR2 and FFAR3 has focused on obesity, diabetes, and digestive system diseases, more and more scientists are exploring the role of these receptors in neurodegenerative diseases, especially PD. A cell experiment confirmed that sodium butyrate can protect dopaminergic cells from Salsolinol-induced neurotoxicity by activating FFAR3 [29]. FFAR2 was also proved to be involved in regulating the growth and maturation of microglia, which will be discussed later [30]. It can be seen that FFAR2 and FFAR3 may have a direct effect on the CNS, but it is controversial that the expression of FFAR2 and FFAR3 in nerve cells is very low and their function is unclear. Interestingly, studies have found that sodium butyrate can provide effective neuroprotection by acting on FFAR2 and FFAR3 expressed in peripheral tissues. SCFAs can combine FFAR2 and FFAR3 expressed in enteric endocrine cells to increase the secretion of glucagon-like peptide-1 (GLP-1), thus alleviating the motor symptoms and dopaminergic neurodegeneration induced by MPTP [31]. Additionally, osteocalcin could play a neuroprotective role in PD mice by increasing the production of propionate which acts as an FFAR3 agonist targeting the enteric nervous system (ENS) [32].

## SCFAs and PD Pathology

It is known that PD is characterized by cerebral pathology. However, since Braak’s staging theory was proposed, people have gradually realized the essential role of the brain-gut axis in PD [33, 34]. As more evidence has emerged that gut microbiota might play a role in the interaction between the gut and brain, the concept of the “brain-gut-microbiota axis” has been proposed. SCFAs are critical for maintaining intestinal function and signal communication of the brain-gut-microbiota axis. Therefore, in this section, we analyzed the role of SCFAs in PD pathology from three dimensions: the gut, brain-gut-microbiota axis, and brain.

### SCFAs and Gut

#### Intestinal Mucosal Barrier

A significant increase in 24-h urine excretion of sucralose was observed in PD patients compared to the control group, indicating an increase in colonic permeability [35]. Another method for assessing intestinal barrier function *in vivo* is to measure α1-antitrypsin and zonulin in feces. In a case-control study, the level of α1-antitrypsin and zonulin in PD patients increased significantly [36], indicating impaired intestinal barrier function in these patients. SCFAs contribute to the stability of the intestinal mucosal barrier in many ways. First, SCFAs provide energy for intestinal epithelial cells. SCFAs can also regulate the expression of tight junction proteins (TJPs) to enhance intestinal mucosal barrier function, through stabilization of hypoxia-inducible factor (HIF) and activation of AMP-Activated Protein Kinase (AMPK) [37, 38]. In addition, SCFAs can enhance the barrier by stimulating mucus production through the activation of FFAR3 [39]. In MPTP mice, propionate improved intestinal mucosal barrier damage and dyskinesia by inhibiting the AKT signaling pathway [40], and butyrate enhanced intestinal barrier stability by activating GPR109A and inhibiting the TLR4/NF-κB pathway [41]. By enhancing the intestinal barrier and reducing the penetration of inflammatory factors, bacterial products and α-synuclein (α-syn) into the systemic circulation, SCFAs will reduce systemic inflammation and neuroinflammation in PD.

#### Intestinal Inflammation

Intestinal inflammation is common in people with PD. Gene expression encoding proinflammatory cytokines in intestinal tissues is increased in PD patients [42, 43]. There were also more CD3+T cells and cells expressing Toll-like Receptor 4 (TLR4). After TLR4 was knocked out in PD mice, symptoms were significantly alleviated [35, 44, 45]. Another piece of evidence is the close association between PD and inflammatory bowel disease (IBD). Previous autopsy studies revealed aggregated α-syn in the ENS of PD patients [46, 47], whereas a recent study discovered α-syn aggregates in the ENS of IBD patients [48]. In addition, IBD patients have a higher risk of PD [49]. The long-term intestinal inflammatory state can induce abnormal α-syn aggregation and aggregation of α-syn can also aggravate intestinal inflammation.

The latest research found intestinal inflammation could trigger gut-to-brain propagation of α-syn and amyloid protein β (Aβ) in a C/EBPβ/AEP-dependent manner [50, 51]. Interestingly, SCFAs could inhibit the increased expression of C/EBPβ and thus further suppress intestinal inflammation [52]. Therefore, the role of SCFAs in regulating intestinal inflammation of PD and their effect on gut-brain transmission of α-syn should be a focus of research. GPR109A signals stimulated by BA can increase interleukin (IL)-18 secretion in intestinal epithelial cells and stimulate the production of Treg cells and IL-10-producing T-cells [53], which is essential for maintaining balance in the intestinal immune environment. In addition, butyrate can inhibit the TLR4/MyD88/NF-B signaling pathway, which is abnormally activated in the brain and gut of MPTP mice [54]. Thus, inhibition of intestinal inflammation by SCFAs is an important mechanism for resisting neuroinflammation in PD.

### SCFAs and the Brain-Gut-Microbiota Axis

#### Gut Microbiota

Significant changes in gut microbiota were found in PD patients, and these changes occurred in the early stage, which may be a precursor biomarker of PD [55, 56]. In addition, dysbiosis of gut microbiota is likely to be a potential trigger factor for PD. Compared to germ-free (GF) mice, the presence of intestinal microorganisms can aggravate motor symptoms and central pathological changes in ASO (α-syn overexpressing) mice, while antibiotic treatment can alleviate these changes [4]. Transplantation of feces from MPTP mice to healthy mice could lead to motor function impairment and a decrease in striatal dopamine levels, whereas transplantation of feces from healthy mice to PD mice could relieve motor dysfunction and restore striatal dopamine levels [57]. Therefore, the gut microbiota is essential for the pathological progress of PD. Analyzing the composition of gut microbiota in PD patients, many studies have demonstrated a decrease in SCFAs-producing bacteria and an increase in proinflammatory bacteria [58, 59]. Interestingly, SCFAs can also affect the composition and structure of gut microbiota by modifying intestinal pH, mucosal permeability, mucin synthesis, and intestinal immunity. Supplementation of sodium butyrate effectively improved gut dysbiosis in rotenone and MPTP models and helped establish a new gut microbiota balance [54, 60]. Therefore, there may be a reciprocal regulatory relationship between microbial metabolites and gut microbiota.

#### Vagus Nerve

The vagus nerve is an important channel for signal transmission and material transport between the brain and intestine. Through activation of the vagus nerve, the changes in gut microbes can affect the brain in terms of behavior and mood. Vagal nerve stimulation is an effective treatment in a variety of PD models, displaying significant anti-inflammatory and neuroprotective effects, but chronic impairment of vagus nerve function led to inhibition of dopamine neurons [61–63]. FFAR3 is found in the afferent nervous system around the portal vein, as well as the intestinal plexus and autonomic nerve [64, 65]. SCFAs in the intestine can stimulate vagal afferent pathways, increasing parasympathetic output from various brain areas and altering the expression in specific brain regions [66]. The infusion of sodium butyrate (10 mM) into anesthetized rats caused vagal afferent discharge, which was eliminated after subphrenic vagotomy [67]. The mechanism of vagal activation by SCFAs in PD deserves further study.

#### Intestinal Endocrine System

The brain-gut interaction is also significantly influenced by the intestinal endocrine system. The intestine is not only a digestive organ, but also the largest endocrine organ. Changes in the intestinal endocrine system will significantly affect the activities of the CNS. After binding FFAR2 and FFAR3 expressed in intestinal endocrine cells, SCFAs will stimulate the secretion of peptide YY (PYY) and GLP-1 [68]. These peptides can be transmitted to the brain through vagal afferents and circulating blood, influencing appetite and eating behavior. Receptors of GLP-1 and PYY are expressed in different brain regions [69, 70]. In addition, these peptides can regulate cognitive and emotional processes, including anti-anxiety and anti-depression effects, as well as the improvement of memory and neural plasticity [70–73]. As described previously, sodium butyrate can increase the expression of GLP-1 in the intestine and GLP-1R in the brain to improve PD in animal models [31]. Therefore, SCFAs can mediate the brain-gut interaction by affecting the intestinal endocrine system, which may have positive implications for PD patients.

#### Systemic Circulation

Besides intestinal inflammation and neuroinflammation, proinflammatory cytokines such as tumor necrosis factor (TNF)-α, IL-1, and IL-6 increased in the peripheral blood of PD patients [74, 75]. The number of activated T cells and NK cells also increased, but naive T cells and IL-17-producing T cells decreased [76–78]. T-cell infiltration and α-syn were found in the CNS and peripheral blood of PD patients, and α-syn is the antigen target of some T cells [79–81]. Evidence suggests a subtle relationship between central inflammation and peripheral inflammation. SCFAs can prevent bacteria and bacterial inflammatory products from migrating into systemic circulation by enhancing the intestinal mucosal barrier. Furthermore, SCFAs can directly interact with immune cells to regulate peripheral immunity. As previously stated, FFAR2 and FFAR3 are abundantly expressed in various immune cells. It was reported that SCFAs could regulate the differentiation, recruitment, and activation of different immune cells [82]. For example, SCFAs can regulate the number and function of peripheral Treg cells and promote immune tolerance by inhibiting HDACs. Through the interaction between immune cells and SCFAs, NF-κB activity was inhibited, and inflammatory cytokines were reduced [83, 84]. These functions are crucial for maintaining balance in the immune environment.

### SCFAs and the Brain

#### Blood-brain Barrier

The blood-brain barrier (BBB) is mainly composed of endothelial cells, astrocytes, and pericytes, embedded with various transmembrane proteins, such as the claudin protein family, occludins, and zonula occluden-1 (ZO-1) [85]. The BBB is highly selective and can prevent harmful toxins and pathogens from entering the brain. The integrity of the BBB is destroyed in PD patients, with decreased expression of tight junction proteins (TJPs) [86, 87]. α-syn aggregation can also increase BBB permeability and accelerate the pathological development of PD [88, 89]. Destruction of the BBB may allow harmful substances and inflammatory factors to enter the CNS, causing inflammation and neuronal damage. Currently, SCFAs have been proven to help maintain the integrity of the BBB. SCFAs can regulate the expression of TJPs through inhibition of NF-κB and activation of Nrf2, a transcription factor involved in the antioxidant pathway [90]. GF mice showed a decrease in the expression of TJPs, which led to an increase in the permeability of the BBB [91]. Interestingly, either GF mice monoclonal by SCFAs-producing strains or GF mice orally gavaged with sodium butyrate effectively reversed the increase in permeability through the increased expression of TJPs. A similar phenomenon has been verified in mice with MPTP-induced PD. The levels of occludins and ZO-1 in the brain were significantly reduced in PD mice but significantly increased in PD mice treated with intragastric sodium butyrate [31]. In addition, SCFAs can maintain the integrity of the BBB by interacting with FFAR3 receptors on brain endothelial cells. Studies have found that propionate can reduce the expression of CD14 on the cell surface and activate Nrf2, thereby reducing the oxidative stress injury of the BBB [28]. These results support the beneficial role of SCFAs in the protection of the BBB.

#### Neuroinflammation

Positron emission tomography revealed that activated microglia widely exist in different brain regions of PD patients, such as the substantia nigra, brainstem, and basal ganglia [92]. Microglia are associated with neuroinflammation during the development of PD due to the consistently elevated levels of proinflammatory cytokines in reactive microglia [93]. In normal conditions, microglia can clear up foreign pathogens and toxins, including aggregated α-syn, but long-term chronic microglial activation can increase neurotoxicity and induce abnormal aggregation of α-syn [94]. The gut microbiota and microbial metabolites appear to be crucial for the growth of the brain’s immune system. Erny *et al*. found that GF mice exhibited hypoplasia of microglia and impaired innate immune response, while the supplementation of a SCFAs mixture promoted the maturation of microglia [30]. Interestingly, this study also discovered that the microglia of FFAR2 knockout mice were severely malformed and showed immature morphology. However, in line with earlier studies, they did not detect FFAR2 mRNA expression in microglia and neurons; therefore, it is still unknown how SCFAs affect the development and maturation of microglia. One year later this question was raised, and the role of SCFAs in the neuroinflammation of PD triggered a new wave of controversy. Most studies have confirmed that SCFAs play an anti-inflammatory role in the periphery, but there are still different opinions on neuroinflammation. Similar to the previous research ideas, Sampson *et al*. expanded the ASO mouse model [4]. Compared with GF-ASO mice, SPF-ASO mice showed more severe motor defects and neuroinflammation. Surprisingly, the administration of a SCFAs mixture to GF-ASO mice promoted motor dysfunction, neuroinflammation, and α-syn aggregation, inducing similar alterations to SPF-ASO mice.

The current controversy is mainly that supplementation with SCFAs under sterile conditions can activate the neuroimmune system and increase neuroinflammation, thereby exacerbating abnormal protein deposition in the brain [4, 95]. However, under SPF conditions, most studies still find that SCFAs play an anti-neuroinflammatory role. SCFAs can directly inhibit inflammatory signal pathways such as NF-κB and MAPK in the CNS, and can also inhibit neuroinflammation by reducing peripheral inflammation and increasing BBB stability. Toxic models of PD, such as MPTP and 6-OHDA, have significant neuroinflammation but are effectively suppressed by supplementation with SCFAs [23, 41, 54, 96]. A prebiotic diet effectively improved the symptoms and pathology of ASO mice by increasing the production of SCFAs to inhibit microglia activation and induce their conversion to a neuroprotective subtype [97]. Interestingly, this study found that the levels of SCFAs and their receptors were not increased in the brain and that histone acetylation levels were not significantly altered, consistent with the reports by Erny [30]. This evidence strongly supports the possibility that SCFAs may indirectly regulate the state of immune cells in the brain *via* the periphery. A chronic cerebral hypoperfusion model showed decreased fecal AA and PA but SCFAs replenishment exerted anti-neuroinflammatory effects by inhibiting microglial activation as well as switching microglial phenotype from M1 toward M2 [98]. Although the exact mechanism is unclear, it is certain that SCFAs have anti-neuroinflammation effects in PD by regulating microglia differentiation and maturation and inhibiting neuroinflammatory pathways through direct or indirect pathways.

#### Damage to Dopaminergic Neurons

The degeneration and loss of dopaminergic neurons is the most important pathological feature related to multiple factors, such as mitochondrial damage, oxidative stress, and cell apoptosis [99–101]. SCFAs can promote the synthesis of tyrosine hydroxylase (TH) to increase brain dopamine levels and reduce oxidative stress and cell apoptosis by inhibiting the NF-κB signaling pathway [102, 103]. SCFAs also increase the expression of GDNF and BDNF in astrocytes, which are important in regulating the growth, survival, and differentiation of neurons and synapses [104, 105]. In addition, SCFAs can increase the expression of DNA repair genes, thereby reducing α-syn-induced DNA damage [102]. Overall, SCFAs play an important protective role in dopaminergic neurons. We also suggest that attention should be paid to the effect of FFAR2 and FFAR3 expressed in neurons on PD. It was found that inhibition of FFAR2 increased Aβ-induced neurotoxicity, and the FFAR2 receptor agonist, Fenchol, could effectively prevent Aβ-induced neurodegeneration in a FFAR2-dependent manner [106]. The possible roles of FFAR2 and FFAR3 in α-syn-induced neurotoxicity also require future attention.

#### α-syn Aggregation and Transmission

Aggregation and propagation of α-syn is a key factor in the progression of PD pathology. SCFAs can affect α-syn aggregation by regulating α-syn expression and neuroinflammation. Early studies found that sodium butyrate could increase the α-syn protein level [107], and PA stimulated the transcriptional activation of α-syn in primary mesencephalic dopamine neurons treated with rotenone [103]. A recent study found that BA treatment increased α-syn transcription, but not the protein level [108]. Further exploration revealed that it was Atg5-mediated autophagy and activation of the PI3K/Akt/mTOR signaling pathway that led to the difference between mRNA and protein levels. Another study found that sodium butyrate could promote PGC-1 to activate the autophagy pathway and reduce α-syn expression by inhibiting HDACs [109]. Neuroinflammation is another important factor in promoting α-syn misfolding and aggregation. SCFAs can reduce the activation of microglia and the production of proinflammatory cytokines to mitigate α-syn-related pathology in the brain.

**Fig. 2:**
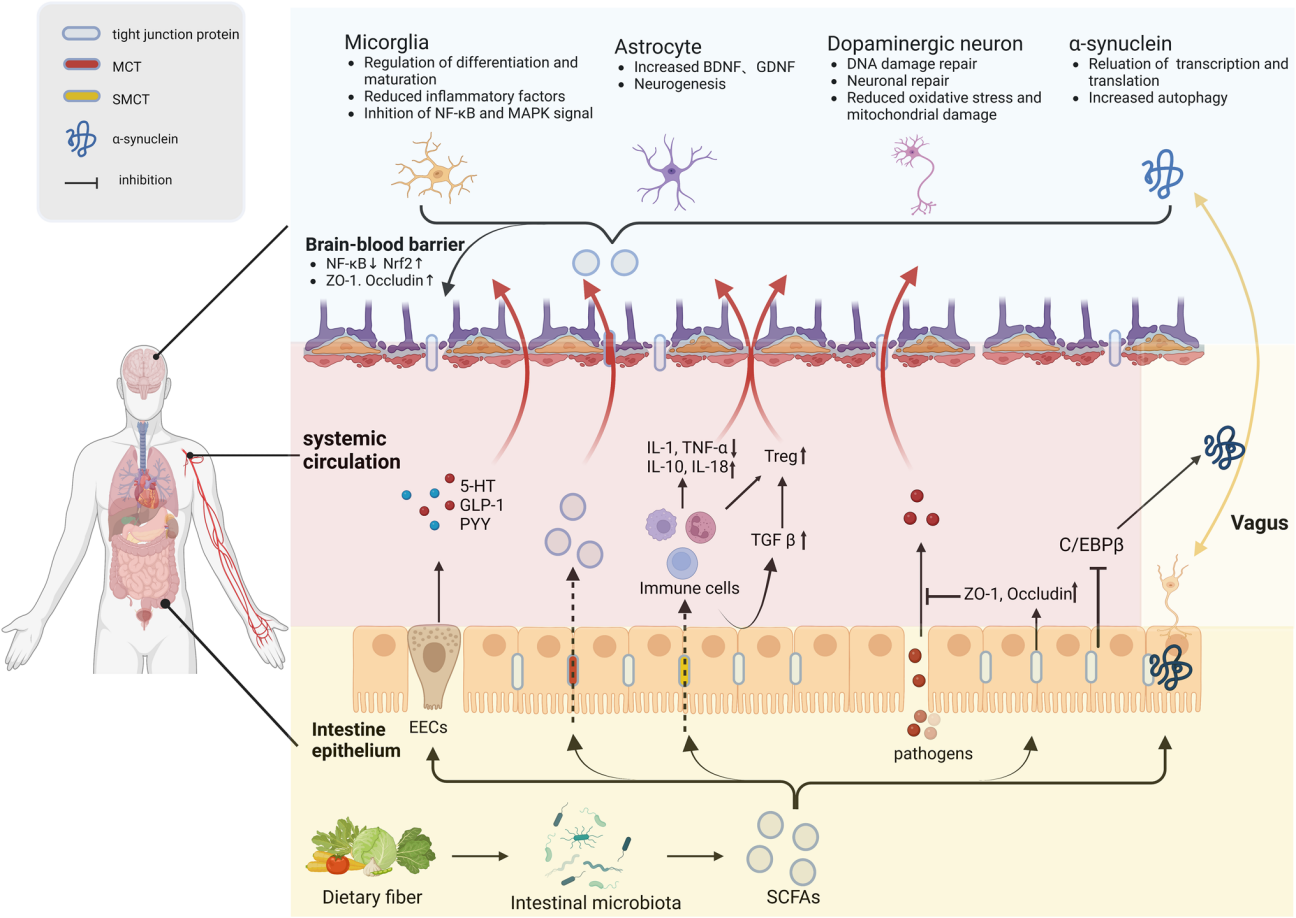
Role of SCFAs in the pathology of Parkinson’s disease. Dietary fiber is fermented by intestinal microbiota to produce SCFAs, which enter intestinal epithelial cells through MCT and SMCT transporters. SCFAs can enhance the integrity of the intestinal mucosal barrier by increasing the expression of tight junction proteins, which help reduce the penetration of pathogens. SCFAs can also inhibit the C/EBP pathway to reduce the spread of α-syn from the gut to the brain. In enteroendocrine cells, SCFAs will promote the secretion of 5-HT, GLP-1, and PYY, which are important neuroprotective factors. In the immune pathway, SCFAs regulate the differentiation and function of immune cells and the secretion of cytokines in the peripheral circulation, indirectly influencing the central immune environment. A small number of SCFAs can also directly cross the blood-brain barrier to play a central regulatory role. The SCFAs entering the brain can enhance the integrity of the blood-brain barrier, regulate the activation of microglia, increase the expression of BDNF and GDNF in glial cells, and modulate the expression and propagation of α-syn. Created with BioRender.com.

Targeted injection of PFF of α-syn provides an effective model for studying the transmission of α-syn. Pathological transmission of α-syn to the brain can be observed after PFF of α-syn was injected into the brain, olfactory bulb, and intestine [110–112]. Excitingly, the administration of sodium butyrate effectively reduced the phosphorylated α-syn content in the substantia nigra, after unilateral injection of PFF into the striatum [113]. SCFAs are involved in maintaining the integrity of the gut barrier, which might affect the transmission of α-syn between the gut and the brain; thus, it is necessary to verify this in the PD model with gastrointestinal injections of PFF. The mechanisms of SCFAs in PD are illustrated in Fig. 2 and relevant basic studies are summarized in Table 1.

**Table 1:** Effects of SCFAs in animal and cellular models of PD

SCFAs	Treatment	PD model	Results	Mechanism
Butyrate	Oral gavage	Rat induced by PFF of α-syn	Motor defect↓, death of dopamine neurons↓, neuroinflammation↓[113]	Inhibition of HDACs–autophagy pathway↑- α-syn pathology↓
	Oral gavage	6-OHDA mice pretreated with CFX	Motor defect↓, neuroinflammation↓, colon injury↓, peripheral inflammation↓, and dysbiosis↓ [114]	Anti-inflammatory and anti-apoptotic effects
	Oral gavage	MPTP mice	Motor defect↓, death of dopamine neurons↓ [31]	GLP-1 in the colon↑, GLP-1R↑ in the brain, blood-brain barrier↑
	Oral gavage	MPTP mice	Motor defect↑, death of dopamine neurons↑, neuroinflammation↑[115]	Oxidative stress↑, intestinal barrier↓, pro-inflammatory effect↑
	Oral gavage	MPTP mice	Motor defect↓, death of dopamine neurons↓, neuroinflammation↓ , α-syn accumulation↓[23]	NF-κB signaling pathway↓, MAPK signaling pathway↓
	Oral gavage	MPTP mice	Motor defect↓, dopamine↑, dopamine neurons↑ , gut dysbiosis↓[54]	TLR4/MyD88/NF-κB pathway↓–intestinal inflammation and neuroinflammation↓
	Oral gavage	MPTP mice	Motor defect↓, dopamine↑, neuroinflammation↓[41]	Activation of GPR109A–NF-κB pathway ↓– intestinal permeability↑
	Oral gavage	Mice induced by rotenone	Motor defect↓, α-syn pathology↓, gut dysfunction↓ [60]	GLP-1↑, microbiota dysbiosis↓
	Intraperitoneal injection	6-OHDA rat	Motor defect↓, striatal dopamine level↑, neuroinflammation↓[96]	Inhibition of HDACs–histone H3 acetylation level↑– BDNF expression↑
	Intraperitoneal injections	6-OHDA rat	Cognitive impairment↓, attention concentration capacity↑[116]	None
	Addition in food	Drosophila induced by rotenone	Motor defect↓, lifespan↑[17]	Inhibition of HDAC–Dopamine↑
	Addition in medium	SH-SY5Y cells treated with salsolinol	Cell viability↑[29]	Activation of FFAR3
	Addition in medium	α-syn expressing LUHMES cells	DNA damage↓[102]	Inhibition of HDACs–histone H3 acetylation level↑– DNA repair gene expression↑
	Addition in medium	PC12 cells treated with rotenone	Cell viability↑, α-syn↓, p-α-syn↓[109]	Inhibition of HDACs–PGC-1α↑– autophagy↑– α-syn expression↓
Propionate	Addition in medium	Primary dopaminergic neurons treated with rotenone	TH+ cells↑, neurite length↑^[103]^	STAT3 signaling pathway↑
	Addition to drinking water	6-OHDA mice	Motor defect↓, death of dopamine neurons↓[32]	Activation of FFAR3
	Oral gavage	MPTP mice	Motor defect↓, intestinal epithelial barrier↑[40]	AKT signaling pathway↓
Mixture	Addition to drinking water	ASO transgenic GF mice	Motor defect↑, neuroinflammation↑, α-syn aggregation↑[4]	Pro-inflammatory role

LUHMES cells, Lund human mesencephalic cells; CFX, Ceftriaxone; PGC-1α, Peroxisome proliferator-activated receptor-γ coactivator-1α

## SCFAs and Non-motor Symptoms of PD

The pathology discussed earlier relates primarily to motor symptoms, PD patients also show many non-motor symptoms, such as constipation, sleep disorders, and depression. Some non-motor symptoms often precede motor symptoms, causing a marked impact on the quality of life and treatment effect of PD patients. Therefore, early identification and treatment of non-motor symptoms will greatly improve the prognosis of PD patients. Some preclinical models also showed significant non-motor symptoms, such as depression and gastrointestinal dysfunction in the 6-OHDA model [117]. Recent evidence suggests a strong association between SCFAs and non-motor symptoms of PD, and SCFAs have potential therapeutic value for various non-motor symptoms.

### Constipation

Constipation is the most common non-motor symptom in PD patients, with a prevalence of around 80% [118]. It is mainly due to impaired cholinergic transmission and α-syn aggregation of the underlying ENS. Compared to WT mice, ASO mice showed a robust expression of α-syn in the ENS and a typical syndrome of constipation characterized by a reduction in fecal water content, fecal pellet output, and prolonged gut transit time [119]. Similarly, human A53T α-syn transgenic mice displayed marked gastrointestinal dysfunction and α-syn pathology in the ENS that preceded motor abnormalities and central pathology by 6 months [120]. Clinical research found that the contents of SCFAs and butyrate-producing bacteria were negatively correlated with the severity of constipation in PD patients [121]. Butyrate could increase the colonic transport speed and the proportion of cholinergic neurons *via* the ENS. This neurochemical plasticity is related to the MCT2 expressed on intestinal neurons and HDACs inhibition [122]. As mentioned previously, SCFAs can enhance the integrity of the intestinal barrier, indicating a potential therapeutic effect on constipation. A recent study also found that sodium butyrate significantly improved the stool frequency and fecal water content of rotenone-induced PD mice [60]. Direct administration of SCFAs to patients is inefficient and impractical, but dietary adjustment, probiotics, and prebiotic fibers all represent potential strategies. When constipated mice were fed acylated starches derived from specific SCFAs for 1 month, it was found that these acylated starches resolved the issue of SCFAs absorption in the small intestine, and acetylated and butylated starch both effectively relieved constipation [123].

### Sleep Disorders

Sleep disorders are very common in the setting of PD, with a prevalence of close to 90% [124]. PD patients exhibit various forms of sleep disorders, such as insomnia, daytime sleepiness, circadian rhythm disorder, and rapid eye movement (REM) sleep behavior disorder (RBD) [125]. Idiopathic RBD (iRBD) is the most dependable hallmark of prodromal PD and the likelihood ratio of iRBD developing into PD is greater than that for the general population [126, 127]. In a clinical study, 16S rRNA sequencing was used to analyze the difference in gut microbiota among healthy people, iRBD, and PD patients. It was found that the number of SCFAs-producing bacteria in iRBD patients was not significantly reduced, while recognized or putative SCFAs-producing genera *Faecalibacterium*, *Roseburia*, and the *Lachnospiraceae* ND3007 group were consistently decreased in PD patients. A decrease in SCFAs-producing bacteria may be a prerequisite for the development of PD [128].

Various studies have demonstrated that SCFAs ameliorate sleep disturbance through immune, neural, and endocrine pathways [129]. Significant alterations in the clock gene were observed both in animal models of PD and in patients [130]. Interestingly, significant rhythmicity was also detected in SCFAs and SCFAs oral gavage, and prebiotic supplementation can facilitate peripheral clock adjustment [131]. Due to the lack of microbial signals, GF mice had an obvious circadian disruption in the liver. Butyrate supplementation significantly increased the ratio of Per2: Bmal1 mRNA in the liver of GF mice [132]. In addition, SCFAs and related bacteria can stimulate the production of 5-HT and melatonin in the intestine [133]; therefore, SCFAs may be closely related to rhythm and sleep. Oral and portal vein injections of sodium butyrate can increase the time of NREM sleep and reduce body temperature by combining with FFAR2 and FFAR3 in the hepatoportal region, achieving the effect of improving sleep [134].

### Depression

**Fig. 3:**
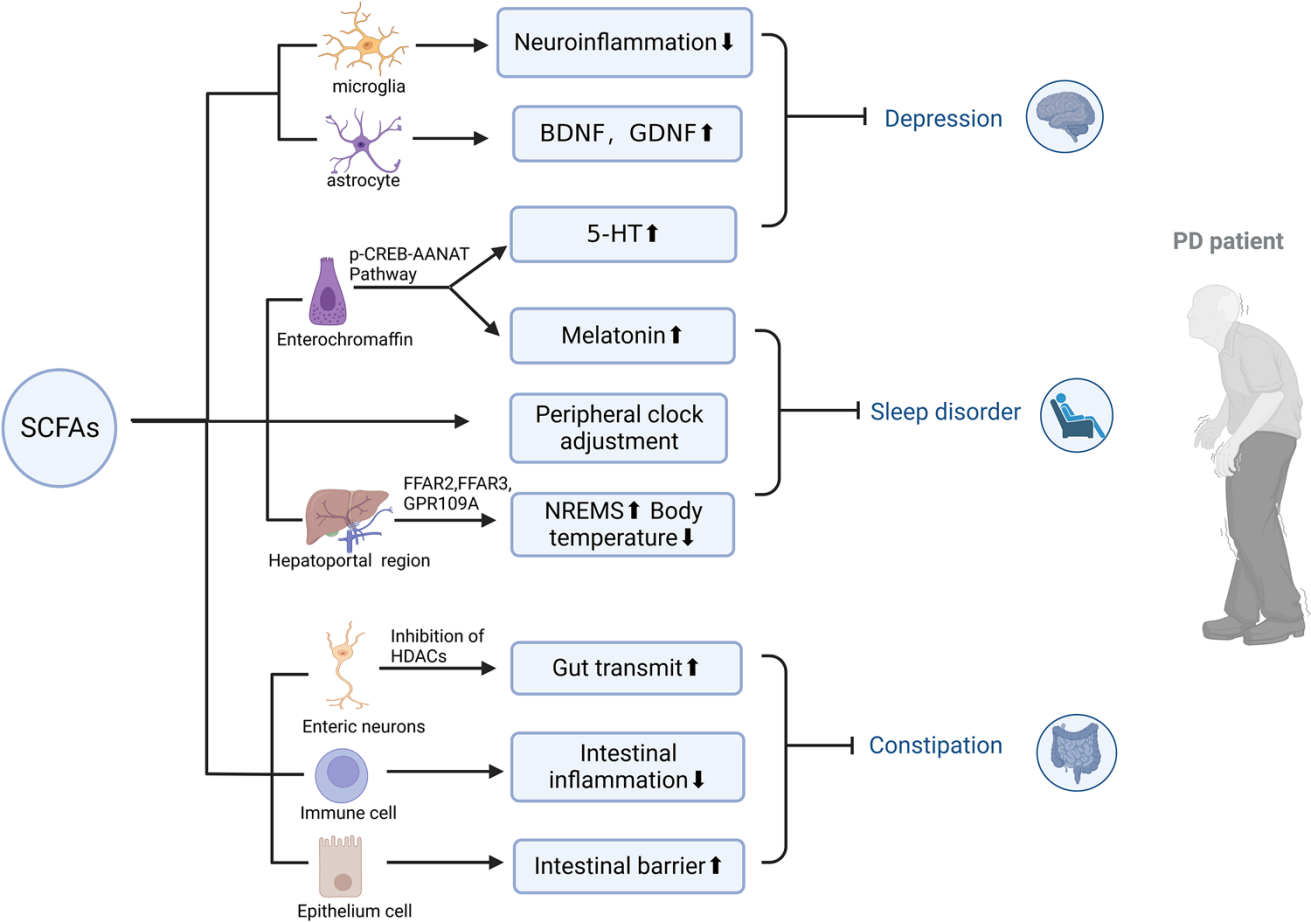
Role of short-chain fatty acids in non-motor symptoms of Parkinson’s disease. SCFAs have potential therapeutic value for non-motor symptoms of PD. SCFAs can promote enterochromaffin cells to secrete 5-HT through the p-CREB-AANAT signal pathway and combine with its role in promoting neurotrophic factors BDNF and GDNF and regulating neuroinflammation, which can effectively improve depressive symptoms. In addition, SCFAs can also promote the secretion of melatonin by intestinal chromaffin cells and regulate the peripheral biological clock. Also, SCFAs can combine with FFAR2 and FFAR3 in the hepatoportal region to release signals to the central nervous system, increase NREMS sleep and reduce body temperature to enhance sleep. Finally, SCFAs can act on intestinal neurons and increase intestinal motility by inhibiting HDACs. Combined with their functions of inhibiting intestinal inflammation and enhancing the intestinal mucosal barrier, they can improve constipation symptoms. Created with BioRender.com.

Depression is the most common neuropsychiatric disorder among people with PD and is related to the degeneration of monoaminergic neurotransmitter systems and fronto-cortical dysfunctions [135]. A case-control study confirmed that low fecal BA content and a decrease in the genera *Roseburia*, *Romboutsia*, and *Prevotella* were related to depressive symptoms in PD patients [136]. A deficiency of 5-HT in the CNS is an important mechanism in depression, but SCFAs can stimulate the production of 5-HT in enterochromaffin cells. Supplementation with SCFAs effectively improved depressive behavior in mice fed with high fructose by preventing the decline in hippocampal nerves and relieving neuroinflammation and BBB damage [137]. Another study also showed that sodium butyrate improved depressive behavior induced by LPS by inhibiting neuroinflammation and oxidative nitrosation stress [138]. In addition, BDNF plays an important role in the survival, maintenance, differentiation, and synaptic plasticity of nerve cells. A reduction in BDNF impaired neurogenesis, resulting in the onset of major depressive disorder, and SCFAs exerted a beneficial effect on depression by recovering the brain’s BDNF level [139] (Fig. 3).

## Clinical Relationship Between SCFAs and PD

Using 16s RNA sequencing and metagenomics, researchers found significant changes in the gut microbiota composition in PD patients. Although the results were not entirely consistent, the general trend showed that *Akkermania*, *Lactobacillus*, and *Enterobacteriaceae* increased in PD patients, while *Prevotella*, *Blautia*, *Coprococcus*, *Roseburia*, and *Faecalbacterium* decreased [140–144]. SCFAs-producing bacteria, especially BA-producing bacteria, were greatly reduced [145, 146]. Unger *et al*. were the first to measure the concentrations of SCFAs in fecal samples from PD patients, and they found that AA, PA, and BA in the feces of PD patients decreased [147]. After that, a series of studies were carried out, and similar conclusions were drawn on the alterations in fecal SCFAs in PD patients. Therefore, there is a consensus that the content of SCFAs in the feces of PD patients decreases.

At present, most studies use fecal concentrations to represent the content of SCFAs produced in the colon. This is indeed a simple and effective alternative, but it should be noted that there are some influencing factors, such as intestinal transit speed, intestinal permeability, and sample handling. For example, faster colonic transit time may lead to reduced absorption of SCFAs and increased SCFAs content measured in feces [148]. In addition, the change in fecal SCFAs content cannot be used to evaluate the content of SCFAs in peripheral circulation or tissues. Therefore, the first case-control study on plasma SCFAs in PD patients has attracted the attention of scientists [149]. This study used gas chromatography to measure the plasma concentrations of SCFAs in 38 PD patients and 33 healthy controls. Before adjusting the covariates, no significant differences were found between the concentrations of AA, BA, and PA in PD patients and the control group, but after adjusting the covariates, the concentrations of AA in PD patients were significantly higher than those in the control group. This was speculated to be related to damage to the intestinal barrier and the leakage of intestinal SCFAs. These results indicate that the plasma SCFAs content may not change in parallel with the fecal SCFAs content. There are some limitations in this study, such as the small sample size and the absence of gut microbiological analysis. However, it is the first study to investigate the relationship between plasma SCFAs content and PD and it provided some new ideas. Based on this, Chen *et al.* investigated the association between the fecal and plasma levels of SCFAs in PD patients [146]. In addition, they analyzed the gut microbiota composition and evaluated its relationship with clinical severity in a large sample of 96 PD patients and 85 healthy controls, making up for the shortcomings of the previous study. Compared with the control group, the feces concentrations of acetate, propionate, and butyrate in PD patients were lower, but the plasma concentrations were higher. Later, Yang *et al*. reached a similar conclusion and found that the combination of fecal and plasma SCFAs could discriminate PD patients from healthy control subjects [121]. In addition, fecal AA and isobutyric acid in PD patients with constipation were lower compared to those without constipation, but plasma AA and PA were higher. Constipation may increase the permeability of the gut-blood barrier in patients with PD. However, different opinions can be found in other studies. Wu *et al*. found that serum PA and BA levels in PD patients were lower than those in the control group [150]. The serum PA level was negatively correlated with motor symptoms and Mini-mental State Examination scores and positively correlated with Hamilton Depression Scale scores. Interestingly, some studies also detected SCFAs in the urine and saliva of PD patients and found that BA in urine was elevated, while AA and PA in saliva were elevated [151, 152]. The relevant clinical research results are summarized in Table 2.

**Table 2:** Alterations in short-chain fatty acids in Parkinson’s disease

Studies	Participants	SCFAs in feces	SCFAs in plasma	Correlation
Unger *et al*. [147]	34 PD, 34 HC	AA↓ PA↓ BA↓	None	BA in feces (−) use of entacapone
Aho *et al*. [153]	55 PD, 56 HC	PA↓ BA↓	None	SCFAs in feces (+) age of PD onset SCFAs in feces (-) total score of NMSS
Tan *et al*. [145]	104 PD, 96 HC	BA↓	None	BA in feces (+) cognitive score BA in feces (−) severity of gait disorder and constipation
Huang *et al*. [40]	17 PD, 17 HC	AA↓ PA↓ BA↓	None	None
Pablo-Fernandez *et al*. [154]	35 PD, 50 HC	AA↓ PA↓ BA↓	None	None
Augustin *et al*. [155]	77 PD, 113 HC	PA↓ BA↓	None	BA in feces (−) colonic transit time
Shin *et al*. [149]	38 PD, 33 HC	None	BA↑	AA in feces (+) age PA in feces (−) UPDRS Part III score and the use of entacapone BA in feces (+) use of monoamine oxidase inhibitors BA in feces (−) use of anticholinergic drugs
Wu *et al*. [150]	50 PD, 50 HC	None	AA↓ PA↓ BA↓	PA in plasma (−) UPDRS Part III score and MMSE score PA in plasma (+) HAMD score
He *et al*. [156]	46 PD, 46 HC	None	PA↑	None
Voigt *et al*. [157]	74 PD, 20 HC	None	LA↓	BA in plasma (−) constipation
Toczylowska *et al*. [158]	19 PD, 21 HC	None	AA↑	None
Qi *et al*. [159]	44 PD, 42 HC	None	AA↑ PA↓	None
Chen *et al*. [146]	96 PD, 85 HC	AA↓ PA↓ BA↓	AA↑ PA↑ BA↑	AA, PA, BA in feces, and PA in plasma (-) UPDRS Part III score BA in plasma (−) MMSE score
Yang *et al*. [121]	95 PD, 33 HC	AA↓ PA↓ BA↓	AA↑ PA↑	Plasma/fecal ratio of SCFAs (+) α1-AT in feces SCFAs in plasma (+) constipation SCFAs in feces (−) constipation AA and BA in feces (−) disease severity AA and PA in plasma + disease severity

AA, acetic acid; PA, propanoic acid; BA, butyric acid; LA, lactic acid; HC, healthy controls; NONE, not measured; UPDRS, Unified Parkinson’s disease rating scale; MMSE, Mini-mental state examination; HAMD, Hamilton depression scale; NMSS, Non-motor symptoms scale

(+) Positive correlation; (−) Negative correlation

In conclusion, the alterations and significance of plasma SCFAs content in PD patients are uncertain. Some other metabolic pathways can produce SCFAs, such as plasma acetate, which can be derived from endogenous products of fatty acid oxidation [160]. Furthermore, SCFAs in plasma cannot directly represent their role in the CNS but are closer to their role in peripheral tissues. Therefore, it is of little significance to simply analyze the content of SCFAs in peripheral blood. We need to jointly analyze the changes of SCFAs in feces and plasma. Most SCFAs originating from the intestine are used for intestinal energy supply and liver metabolism, and only a few SCFAs enter the peripheral circulation. If it is determined that the plasma SCFAs content in PD patients increases and the fecal SCFAs content decreases, it may reflect the increased permeability of the intestinal mucosal barrier or dysfunction of the liver, which causes the “leak” in SCFAs.

## Discussion

Due to the significant changes in SCFAs that occur in PD and the protective effect of SCFAs on the nervous system, a number of scholars have proposed the view that alterations in SCFAs are responsible for the pathogenesis of PD. From existing evidence, however, this view is somewhat exaggerated. Changes in SCFAs and SCFAs-producing bacteria are not disease-specific. Reductions in fecal SCFAs can also be observed in IBS, chronic kidney disease, and other neurodegenerative diseases [161–163]. In addition, the distribution of SCFAs and their receptors in the brain is relatively low, and there is a lack of strong evidence to prove the direct regulatory effect of SCFAs on specific neurons. In particular, a clear explanation for the causal relationship between SCFAs and specific pathological features of PD such as the loss of dopaminergic neurons and the formation of a Lewy body, is still lacking. Alterations in SCFAs are more likely to be secondary to dysbiosis of gut microbiota in the early stages of PD. The reduction in SCFAs-producing bacteria and destruction of the intestinal barrier leads to a decrease in fecal SCFAs and an increase in plasma SCFAs. This secondary change, in turn, exacerbates intestinal inflammation and systemic immune disorders, as well as damage to the BBB, leading to the infiltration of peripheral immune cells, toxins, and cytokines. A reduction in SCFAs also leads to abnormal brain-gut interaction, indirectly affecting the immune status and neuronal function within the brain. We think it necessary to elucidate the relationship between SCFAs and the aggregation and spread of α-syn in the gut, as both SCFAs deficiency and intestinal α-syn aggregation are early alterations in PD. Their causal relationship is key in proving a direct link between SCFAs and PD.

In addition, it must be emphasized again that the relationship between SCFAs and neuroinflammation requires further clarification. Although we concluded that SCFAs remain predominantly neuroprotective in the presence of intestinal flora, there are studies with contradictory findings. In particular, one study found that oral administration of sodium butyrate to MPTP mice under SPF conditions surprisingly increased neuroinflammation and increased the loss of dopamine neurons [115]. Paradoxical results were also observed in models of Alzheimer’s disease, amyloidosis, and neuropathic pain [95, 164, 165]. Differences in the effect of SCFAs on neuroinflammation are related to many factors, such as microbiological control levels for laboratory animals, the method of administration, mixture or single drug administration, and the drug concentration. Future research should explore the effects of different types and concentrations of SCFAs on the immune function of the brain in both germ-free and conventional environments, and determine the optimal doses and types to promote brain health and immune function. Clarifying the role of SCFAs in the neuroimmune system under physiological conditions is a prerequisite for understanding their effects on neuroinflammation in PD.

To date, there is still no effective treatment for PD, and symptomatic treatment is still the main therapeutic option. Therefore, early diagnosis and treatment are crucial. Early changes in SCFAs combined with other more specific diagnostic aids, such as real-time vibration-induced protein amplification with high sensitivity and specificity for abnormally folded α-syn, can be used to identify fibrillar α-syn in biological fluids [166], as well as in conjunction with early detection of prodromal symptoms, such as RBD, to better diagnose PD at an early stage. Direct injection or oral administration of SCFAs as treatment is unreasonable and unrealistic in clinical practice. Therefore, SCFAs-producing probiotics and prebiotics are very promising alternatives, as they could effectively avoid the absorption of SCFAs in the upper digestive tract, which will better simulate the absorption and action mode of SCFAs *in vivo*. Prebiotics with specific chemical structures can be selected or designed to achieve an increase in the targeting of SCFAs and SCFAs-producing bacteria in the colon [167]. Many probiotics and prebiotics have been proven to be effective in improving PD by increasing the production of SCFAs. In MPTP mice, oral administration of *Bifidobacterium breve* CCFM1067 protected the blood-brain and intestinal barriers from damage by improving intestinal microecology and increasing the synthesis of SCFAs [168]. In ASO mice, a prebiotic diet modulated the activation of microglia and motor deficits by altering gut microbiome composition and the content of SCFAs [97]. Synbiotics are compound preparations of probiotics and prebiotics, which can more effectively exert the physiological activity of probiotics. Polymannuronic acid (PM) and *Lacticaseibacillus rhamnosus GG* (LGG) in combination were found to have much better neuroprotective effects on PD than PM or LGG alone [169], indicating the therapeutic potential of synbiotics in PD. Also worthy of attention are medicinal plants, such as *Mucuna pruriens* (Mp) and *Withania somnifera* (Ws), which are rich in crude protein, essential fatty acids, and starch. They not only promote the production of SCFAs, but also have many bioactive components, such as ursolic acid in Mp and chlorogenic acid in Ws, and both showed potent anti-Parkinsonian activity in a toxin-induced Parkinsonian mouse model [100, 170].

## Conclusion

In this review, we comprehensively summarize the relationship between SCFAs and PD from pathology to the clinic. Significant alterations in SCFAs are present in patients with PD and are closely associated with motor symptoms and non-motor symptoms. SCFAs affect the pathological progress of PD through multiple dimensions. In the future, more attention should be paid to the diagnostic and therapeutic value of SCFAs for PD. There are still some problems that need to be solved urgently. First, we need to clarify the regulatory mechanism of SCFAs on microglia. Direct central regulation or indirect peripheral regulation? Second, the decrease in fecal SCFAs in PD patients is now a consensus, but the significance and alteration of plasma SCFAs need to be further confirmed, including in other tissues. Third, it is necessary to identify a stable and highly recognized PD model with specific non-motor symptoms to verify the therapeutic potential of SCFAs in non-motor symptoms. Fourth, the value of SCFAs-producing probiotics, prebiotics, and synbiotics in PD deserves further development. Some shortcomings in this review should also be noted. The study of SCFAs-producing bacteria in PD has not been summarized. In addition, we did not focus on the interaction between SCFAs and other metabolites. It is hoped that future research and reviews will add to these aspects.
